# Liposomes for Cancer Theranostics

**DOI:** 10.3390/pharmaceutics15102448

**Published:** 2023-10-11

**Authors:** Donald A. Fernandes

**Affiliations:** Independent Researcher, Toronto, ON, Canada; dfe.anthony@gmail.com

**Keywords:** theranostics, nanoparticles, therapy, imaging, image-guided therapy

## Abstract

Cancer is one of the most well-studied diseases and there have been significant advancements over the last few decades in understanding its molecular and cellular mechanisms. Although the current treatments (e.g., chemotherapy, radiotherapy, gene therapy and immunotherapy) have provided complete cancer remission for many patients, cancer still remains one of the most common causes of death in the world. The main reasons for the poor response rates for different cancers include the lack of drug specificity, drug resistance and toxic side effects (i.e., in healthy tissues). For addressing the limitations of conventional cancer treatments, nanotechnology has shown to be an important field for constructing different nanoparticles for destroying cancer cells. Due to their size (i.e., less than 1 μm), nanoparticles can deliver significant amounts of cancer drugs to tumors and are able to carry moieties (e.g., folate, peptides) for targeting specific types of cancer cells (i.e., through receptor-mediated endocytosis). Liposomes, composed of phospholipids and an interior aqueous core, can be used as specialized delivery vehicles as they can load different types of cancer therapy agents (e.g., drugs, photosensitizers, genetic material). In addition, the ability to load imaging agents (e.g., fluorophores, radioisotopes, MRI contrast media) enable these nanoparticles to be used for monitoring the progress of treatment. This review examines a wide variety of different liposomes for cancer theranostics, with the different available treatments (e.g., photothermal, photodynamic) and imaging modalities discussed for different cancers.

## 1. Introduction

Nanomaterials have shown to be effective systems for a wide variety of biomedical applications, and have been used for treatment [1], delivery of therapeutics [2] and bio-imaging [3]. Significant developments have arisen in nanotechnology, focusing on nanoparticles for both imaging and therapy, referred to as theranostics [4,5,6,7]. Nanoparticles are advantageous due to their physical and chemical properties, enabling them to be used for tumor monitoring and treatment. Materials such as gold nanoparticles [8,9], quantum dots [10,11], magnetic nanoparticles [12,13], upconversion nanoparticles [14,15], nanoemulsions [16,17,18], nanogels [19,20], micro/nano-bubbles [21,22,23], carbon nanoparticles [24,25], polymeric nanoparticles [26,27] and micelles [28,29] have all successfully been used for the diagnosis and treatment of cancer. Liposomes are unique nanoparticles that have been used for cancer theranostics, due to their ability to load both hydrophobic (i.e., in their lipophilic shell) and hydrophilic therapeutic and imaging agents (i.e., in the aqueous core). The advantages of liposomes include the high loading efficiency of agents, high stability in biological conditions and controllable release kinetics due to stimuli responsiveness and biocompatibility, providing better pharmacokinetics and biodistribution of theranostic agents than many other carriers [30]. 

This review article focuses on the different types of therapies and imaging techniques that are available and that have been reported when using liposomes for cancer theranostics. The different types of liposomes are presented, with a focus on the results from the treatment and imaging of different kinds of cancers. The goal is to highlight the benefits of using liposomes and the variety of treatment and imaging options available for cancer theranostics. 

## 2. Common Cancer Therapies

A wide variety of therapies are available when using nanoparticles (e.g., liposomes) for cancer treatment, depending on the stimuli (i.e., ionizing or non-ionizing) used for excitation and the mechanism for cancer cell death (e.g., apoptosis, ablation). Each treatment option has unique advantages that can be attractive when using specific types of nanoparticles, as well as disadvantages limiting their widespread use (Table 1). Common therapies that can use nanoparticles for improving treatment outcomes include chemotherapy, gene therapy, immunotherapy, photothermal therapy, photodynamic therapy, magneto-thermal therapy, ultrasound responsive therapy and radiotherapy. 

### 2.1. Chemotherapy

Biocompatible and biodegradable nanoparticles such as liposomes can be used for the delivery of chemotherapeutic agents (e.g., paclitaxel, docetaxel, doxorubicin, celecoxib) [31]. Drug-loaded nanoparticles reduce systemic toxicity due to slow drug release rates, increasing the amount of therapeutic agents that can accumulate in tumor tissue (i.e., through the enhanced permeability and retention effect, EPR) [32,33,34,35]. Resistance to chemotherapy remains a significant issue in oncology, reducing its efficacy in treating metastatic tumors. The drug resistance mechanism is highly intricate and various factors reduce the effectiveness of therapeutic agents in inhibiting tumor growth. Drug transporters are responsible for drug efflux, resulting in low therapeutic doses [36,37], while gene mutations, genomic instability, epigenetic alterations (e.g., DNA methylation, protein acetylation), suppression of apoptotic signaling, and overexpression of anti-apoptotic molecules contribute towards multi-drug resistance (MDR) [38]. To reverse MDR (i.e., resistance to anticancer drugs), conventional strategies include the development of inhibitors for ABC transporters (e.g., monoclonal antibodies, compounds), using high doses of chemotherapy, targeting specific messenger RNAs (mRNAs) for rendering MDR genes ineffective and the development of chemotherapeutics that are not substrates of P-glycoprotein or P-gp (i.e., a MDR drug efflux protein) [39]. To better combat multi-drug-resistant cancer, various nanoparticles have been developed, including those containing inhibitors of drug efflux transporters (e.g., tyrosine kinase inhibitors, small interfering RNA) that block function or silence the MDR mRNA [40]. 

### 2.2. Gene Therapy

Nanoparticles such as liposomes can be used for the delivery of genetic material such as plasmid DNA, mRNA, microRNA (miRNA), small interfering RNA (siRNA), short hairpin RNA (shRNA), and antisense oligonucleotides [41,42]. There are several mechanisms by which gene therapies work, for example by replacing a cancer-causing gene with a healthy copy of the gene and/or inactivating a non-functional cancer-causing gene. Replacement of genes can be accomplished by gene transduction, stability maintenance and complete gene expression, or by the correction of gene mutations into its wild-type form [43]. Targets for gene replacement therapy include tumor suppressor genes (e.g., TP53, P21, PTEN). Gene silencing on the other hand involves introducing siRNA or shRNA in tumor cells for targeting a specific complementary sequence to mRNA for its degradation or by blocking the synthesis of proteins [44]. Targets for gene silencing therapy include drug resistance oncogenes (e.g., cMYC, KRAS). Antisense therapy uses antisense oligonucleotides to target mRNA for the downregulation of gene expression (e.g., that are associated with regulating apoptosis, cell growth, metastasis, and angiogenesis) [45]. The miRNA-targeted therapy involves restoring levels of miRNAs that have been altered, using miRNA-duplexes to replace levels of under-expressed miRNAs, or siRNA complementary to the seed sequence of the miRNA of interest [46,47]. Genes can also be introduced, for example in suicide gene therapy, encoding a cytotoxic protein for cancer cell death [48]. In addition, genome editing therapy has recently gained much attention for being able to modify intracellular DNA in a sequence-specific manner (i.e., by insertion, deletion, integration or sequence substitution), using nucleases such as zinc finger nucleases (ZFN), transcription activator-like effector nucleases (TALEN), meganucleases, and CRISPR/Cas9 systems [49,50]. 

### 2.3. Immunotherapy

Immunotherapy and the idea of boosting antitumor activity using the immune system (i.e., via tumor-specific or non-specific immune activation) has established itself as an effective therapy option [51]. For example, a promising and emerging strategy to treat both solid and hematological malignancies is programmed cell death protein 1 (PD-1)/programmed cell death ligand 1 (PD-L1) blockade immunotherapy [52]. This therapy is used to wake the immune system from the suppression of activities, leading to the death of cancer cells by T cells. This is an attractive alternative to treatment using chemotherapeutic agents, which can lead to multi-drug resistance by cancer cells through genetic mutations. Nanoparticles such as liposomes can be used as platforms for the delivery of immune modulators, targeting tumor-associated cells (e.g., dendritic cells, T cells, tumor cells, natural killer cells, and macrophages) for enhancing immunological responses and immunotherapy efficacy [53,54]. The group of proteins and drugs (e.g., cytokines, thalidomide, imiquimod) that are used for immunotherapy mainly target pathways that decrease or increase the amount of certain proteins for enabling the immune system to work optimally for the treatment of cancer. Different types of nanocarriers (i.e., <1 μm) can be developed for immunomodulation by carrying specific immunotherapeutic agents, based on inorganic and organic moieties [55]. 

### 2.4. Photothermal Therapy

Photothermal therapy (PTT) is a therapeutic strategy wherein near-infrared (NIR) light is used to resonate electrons in nanoparticles for producing heat and ablating malignant tumors [56]. When the local temperature from the heat generated by nanoparticles is in the range 43–50 °C, apoptosis in cancer cells can be induced [57]. If cancer cells are exposed for more than 15 min in this temperature range, irreversible tissue damage can occur, with rapid protein denaturation at temperatures above 60 °C (e.g., from thermal tumor ablation) [58]. To improve the optical properties through surface plasmon resonance (i.e., an electromagnetic response that occurs when plasmons are oscillating with the same frequency on the surface of a material), various elements are used for constructing particles, such as gold, silver and carbon [59,60]. Recently, polymer-based nanoparticles have gained attention because of their biocompatibility and biodegradability and use in different types of treatments with high photothermal conversion efficiencies [61,62].

### 2.5. Photodynamic Therapy

Photodynamic therapy (PDT) is a minimally invasive treatment for cancer therapy [63]. Light (i.e., usually NIR) is absorbed by photosensitizer molecules (i.e., loaded in nanoparticles for reducing systemic toxicity), after accumulating in tumor tissues. Photosensitizers are important due to their ability to induce chemical and physical alterations of other molecules, upon absorption of light. The excited photosensitizer can undergo electron and/or energy transfer reactions to produce reactive oxygen species (ROS). These generated ROS can interfere with important molecules (e.g., DNA) and proteins, inhibiting their function and activating certain mechanisms such as apoptosis due to generated oxidative stress [64]. Like PTT, PDT treatment only targets the areas under illumination, minimizing toxicity in healthy cells [65]. Since photosensitizers can also provide fluorescence under laser irradiation, they are loaded in many types of nanoparticles including liposomes for cancer theranostics [66,67,68]. 

### 2.6. Magneto-Thermal Therapy

Magnetic nanoparticles can be used to generate heat using an alternating current (AC) magnetic field for cancer therapy. Magnetic heating (i.e., hyperthermia from magnetically mediated heating of low-frequency electromagnetic waves) and a temperature-responsive or thermally-rupturable layer (i.e., thermosensitive and disrupted above a certain temperature) are two fundamental features required for magneto-thermal delivery and cancer theranostics [13,69,70,71]. Such properties enable therapeutic agents to be released at high quantities at the tumor site after tumor accumulation and heating using magnetic fields, reducing off-target effects (i.e., in other non-cancerous tissues). A common delivery mechanism for magnetically triggered therapies involves the integration of iron oxide nanoparticles (IONPs) in liposomes. Recent developments have involved magneto-thermal therapies (i.e., magneto-photothermal therapy) using magnetic particles with light absorbing molecules/particles and combining NIR lasers and alternating magnetic field induction for improving cancer treatment [72,73,74].

### 2.7. Ultrasound Responsive Therapy

Ultrasound can be used for temperature-sensitive nanosystems such as liposomes, where the encapsulated payload can be released locally through mechanical effects (e.g., cavitation, strain) [75,76,77,78,79]. Ultrasound responsive therapy provides an attractive mechanism for the delivery of therapeutic agents, controlling the release with no degradation of molecules [80]. Other types of nanoparticles/bubbles that require ultrasound for cancer therapy include polypyrrole hollow microspheres [81], microbubbles [82,83,84,85,86], biodegradable poly(methacrylic acid)-based nanocapsules [87], superparamagnetic iron oxide acoustic droplets [88], crown-ether-coated core/shell nanoparticles [89], polymer-grafted mesoporous silica nanoparticles [90], and echogenic glycol chitosan nanoparticles (i.e., that are able to generate ultrasound signals from acoustic cavitation of the resulting bubbles formed) [91].

### 2.8. Radiotherapy

Radiotherapy (RT) using nanoparticles and ionizing radiation (i.e., high energy X-rays or other particles) can cause significant damage in tumor tissue. Nanoparticles such as liposomes serve as radiosensitizers (RSs), containing high atomic number and electron-dense elements for enhancing therapeutic sensitivity [92,93]. High atomic number and electron-dense elements are required for improving the relative dose accumulation of tumors (i.e., for increasing the amount of free radicals and enhancing DNA damage) by enhancing the absorption cross-section of X-rays. A main disadvantage of RT is the dose delivery efficiency in destroying cancer cells, which depends on the tolerance level of normal tissues near tumor tissues. 

## 3. Imaging Modalities Available for Cancer Theranostics

Nanoparticles such as liposomes can load imaging molecules (e.g., radioisotopes, fluorophores, superparamagnetic particles, acoustic scatterers) for enhancing contrast in cancer imaging. Due to the hydrophobic and hydrophilic nature of liposomes, many molecules with varying solubility can be loaded. This makes it possible for liposomes to be used with different imaging modalities such as positron emission tomography, single-photon emission computed tomography, computed tomography, magnetic resonance imaging, optical imaging, ultrasound imaging and photoacoustic imaging, improving the overall benefits and avoiding the limitations of certain imaging techniques (Table 2). 

### 3.1. Positron Emission Tomography 

Positron emission tomography (PET) is routinely used clinically for whole-body imaging, based on visualizing and quantifying positron-emitting radionuclides. These radionuclides are used for emitting pairs of γ-rays for generating imaging contrast and include atoms such as ^11^C, ^13^N, ^15^O, ^18^F, ^44^Sc, ^62^Cu, ^64^Cu, ^68^Ga, ^72^As, ^74^As, ^76^Br, ^82^Rb, ^86^Y, ^89^Zr, and ^124^I [94,95,96,97,98,99,100,101,102]. Because radionuclides are small and are limited by their quick clearance, PET probes can be conjugated to or encapsulated within nanoparticles for improving biodistribution and accumulation at the target site. PET imaging provides high sensitivity (i.e., radionuclide concentrations as low as (sub-)picomolar range) and high signal-to-noise ratios, providing unlimited penetration depth. Disadvantages associated with PET imaging include the lack of anatomical information, the relatively low spatial resolution and the necessity for using radioactive probes, which can be toxic to healthy cells. Despite this, PET imaging can be combined with other imaging modalities such as computed tomography (i.e., PET-CT) and magnetic resonance imaging (i.e., PET-MRI) for overcoming the disadvantages of CT or MRI. Various studies have reported on the use of liposomes with PET imaging (i.e., with radionuclides incorporated) [103] for enhancing tumor accumulation and improving pharmacokinetic properties for cancer theranostics [104]. 

### 3.2. Single-Photon Emission Computed Tomography

Single-photon emission computed tomography (SPECT) imaging uses the non-coincident γ-rays generated by radionuclides such as ^99m^Tc, ^111^In, ^123^I, ^125^I, and ^201^Tl. Because SPECT is analogous to PET, it shares advantages and disadvantages (i.e., previously mentioned for PET). The sensitivity of SPECT is about an order of magnitude lower than that of PET and quantification is somewhat more difficult. In addition, compared to PET where all the emitted photons have the same energy, the energies from radionuclides in SPECT are often different, enabling the assessment of different radiotracers/radiolabeled nanoprobes at the same time. SPECT can be combined with other imaging modalities such as CT (i.e., SPECT/CT) and MRI (i.e., SPECT/MRI) for monitoring tumors and assessing biodistribution using liposomes [105,106,107]. 

### 3.3. Computed Tomography 

X-ray computed tomography (CT) imaging is a modality that uses computer processed X-ray scans to produce tomographic images and 3D visualization of tumors. Contrast images are a result of distinctions in X-ray absorption and attenuation by different components of the body, which can be enhanced by the use of nanoparticles. CT contrast agents have large atomic weight (i.e., high Z value) elements such as iodine (non-radioactive), gold, platinum, bismuth, tantalum, and ytterbium. High atomic number element containing liposome contrast agents for CT, especially CT-based multimodal imaging liposomes, have been used for increasing circulation lifetime and enhancing the accumulation at the tumor site [108,109,110]. CT imaging can provide high spatial resolution with unlimited tissue penetration. However, a relatively high dose of ionizing radiation is required for CT imaging to be effectively used for cancer theranostics. 

### 3.4. Magnetic Resonance Imaging

MRI is a clinically applied imaging modality, which depends on the spin–lattice relaxation and the spin–spin relaxation time of protons contained in different tissues or organs, with the ability to enhance image contrast using nanoparticles (e.g., liposomes) [111,112,113,114,115]. MRI does not use any radiation and can provide excellent anatomic detail and high spatial resolution. Paramagnetic ions, such as manganese (Mn^2+^), iron (Fe^3+^), and gadolinium (Gd^3+^), are usually used to provide MRI contrast. The nanoparticles can alter the spin–lattice and/or spin–spin relaxation time of protons for providing T_1_ and/or T_2_ contrast [116]. PET, SPECT and optical imaging can be used with MRI to improve sensitivity and image resolution, while the addition of CT improves temporal resolution [117]. Although MRI can be used for (pre-) clinical diagnosis and therapy monitoring, it has several disadvantages such as relatively low contrast agent sensitivity, difficult quantification procedures, and significant time and cost involved.

### 3.5. Optical Imaging

Optical imaging (OI) is a non-ionizing, non-invasive imaging modality based on the optical characteristics of tissue components (i.e., from light emission/absorption), which can be enhanced through the introduction of nanoparticles (e.g., liposomes with fluorescent probes and/or upconverting nanoparticles, UCNPs) [118,119]. Biomedical optical imaging can be used for quantitative measurements in real time, providing a wide range of image resolutions for cancer diagnosis and monitoring of treatment. Advantages of OI include the simplicity of use, simultaneous detection of multiple markers, and wide spatial scales ranging from subcellular structures to tissues. However, a main disadvantage of OI is that it cannot be used for deeply penetrating tumors due to limited penetration depth. Compared to conventional fluorophores, UCNPs emit higher-energy visible light when excited by NIR light and can vary in composition for use in multiple imaging modalities. Even though an absolute quantification of accumulation of nanoparticles in tumors can be made using 3D fluorescence molecular tomography (FMT), diffusive scattering of fluorescence emission in the body and strong light absorption by organs and tissues limits the assessment of the biodistribution. 

### 3.6. Ultrasound and Photoacoustic Imaging

Ultrasound imaging (USI) is based on the principle that back-scattered signals from acoustic waves vary depending on the reflection by different tissues, as well as by US contrast agents. USI is a versatile technique, providing a clear depiction of the area of interest, with high temporal and spatial resolution. Particles such as nanoscale liquid–liquid nanoparticles, gas–liquid nanoparticles, and solid nanoparticles have also been reported to contribute towards enhanced US contrast and therapy [120,121,122]. Despite this, USI is limited by relatively low resolution and sensitivity, with a penetration depth that depends on the US frequency used. An imaging modality that is commonly used alongside USI imaging is photoacoustic imaging (PAI). PAI is commonly used for the illumination of light-absorbing molecules and nanoprobes [123,124] in tissues using pulsed laser light for providing signals based on energy absorption, heat generation, and thermoelastic expansion from particles and tissue [125,126,127]. The expansion can be detected using ultrasound detectors, with signals detected at deeper depths and with higher sensitivity when USI is combined. 

## 4. Biomedical Applications of Cancer Theranostic Liposomes

A variety of liposomes have been developed for therapy and imaging of different types of cancers. Liposomes can be produced that either passively (i.e., through EPR effect and accumulation) or actively (i.e., through ligand–receptor interactions) target cancer cells. Both kinds of liposomes have been shown to be effective, enhancing pharmacokinetics (e.g., half-life) for cancer treatment. In addition to promising in vitro and in vivo results, many liposomes are undergoing or have undergone different clinical phase trials (Table 3). 

### 4.1. Breast Cancer

Theranostic dual-layered gold (Au)-containing liposomes can be developed for effective tumor targeting and photothermal therapy [128]. A liposomal layer is added to Au-coated liposomes (i.e., ALs) for producing dual layered NPs (i.e., liposomal ALs called LALs). The NPs are functionalized with polyethylene glycol (PEG) for improving the in vivo stability with radioisotopes (i.e., ^64^Cu-LAL) used for enabling in vivo PET imaging. The addition of Au NPs contributes to PTT due to the excellent photothermal conversion efficiency (i.e., due to surface plasmon resonance phenomenon) and tunability of the absorption band [129,130,131]. Transmission electron microscopy (TEM) was used to visualize the NPs, confirming the decoration of Au NPs on liposomes for ALs and the outer liposomal layer over ALs (i.e., in samples containing LALs). The sizes determined using TEM for ALs and LALs were 61.02 ± 29.22 nm and 72.84 ± 22.49 nm, respectively. The zeta potential decreased from −12.0 mV to −23.7 mV after Au coating on liposomes. The zeta potential increased to −17.7 mV after AL was covered with a liposomal layer, due to the PEG moiety. The LALs were stable in physiological solutions (i.e., deionized water, phosphate-buffered saline or PBS, cell media with 10% fetal bovine serum), with the sizes maintained (i.e., ranging from 60 to 80 nm) over a period of 14 days. Both ALs and LALs have a broad absorbance band with a peak at 800 nm. Under 808 nm laser irradiation with 1 W intensity, the temperatures from solutions containing ALs and LALs increased from ~25 °C (i.e., before laser irradiation) to ~42 °C and ~44 °C, respectively, after 40 min of irradiation (i.e., NPs containing the same Au concentration, ~24 μg/mL). Furthermore, both ALs and LALs were stable in terms of temperature elevation during four repeats of laser irradiation (i.e., on/off cycles), suggesting that the NPs can be irradiated multiple times for PTT with a single injection of NPs. The calculated photothermal efficiency values of ALs and LALs were 34.13% and 37.46%, respectively, which are higher than the values from other photothermal nanomaterials [132,133,134,135,136,137]. In vitro experiments in 4T1 breast cancer cells were carried out to determine the biocompatibility of NPs (i.e., without laser irradiation) and cancer cell viability from PTT using ALs and LALs. The cytotoxicity (i.e., from NPs only) was not significant, showing over 80% survival of cells (i.e., up to Au concentrations of ~12 μg/mL). However, when 4T1 cancer cells are treated with AL and LAL (i.e., both with ~12 μg/mL Au concentration) and laser irradiation (i.e., 2 W/cm^2^, 5 min), the cell viability is as low as ~30% for both types of NPs (i.e., ALs, LALs). The excellent PTT effect of NPs is due to the high rate of cellular uptake of NPs, DNA damage and temperature change from laser irradiation. To demonstrate the ability to be used for in vivo PET imaging, ^64^Cu-LAL (i.e., 4.74 μg of Au) was used. The tumor uptake of NPs increased gradually with time, with the percent injected dose per gram of tumor tissue (ID/g) up to ~16.4%, after 24 h from injection. This is much higher than other nanoparticles used for PTT, such as bare AuNPs (i.e., ~1.6% ID/g) and modified AuNPs (i.e., 5–10% ID/g) after 12–48 h from intravenous injection [138,139,140,141,142,143,144]. Using 808 nm laser irradiation on each tumor site with 2.5 W/cm^2^ (i.e., two times treatment, 24 and 48 h after intravenous injection), the tumor growth inhibition rate of each group was as follows: normal saline + laser was 12.2%, ALs was 14.2%, ALs + laser was 20.4%, LALs was 7.52%, and LALs + laser was 79.4%. This shows that the NPs (i.e., especially LALs) can effectively treat tumors through PTT, while being able to be used for cancer imaging.

Iron oxide nanoparticles (i.e., synthesized using a modified co-precipitation method) and chemotherapeutic drug doxorubicin (DOX) can be passively encapsulated into PEGylated liposomes (i.e., forming magnetoliposomes) [145]. Superparamagnetic iron oxide NPs (i.e., magnetite (Fe_3_O_4_), maghemite (γ-Fe_2_O_3_)) can be used for different biomedical applications [146] such as drug delivery [147], MRI [148] and hyperthermia [149]. Dextrose (Dex) coating around magnetic nanoparticles (MNPs) was confirmed using Fourier transform infrared (FTIR) spectroscopy, with a peak at 523 cm^−1^, shifted from 582 cm^−1^ (i.e., from vibration of Fe–O from MNPs) due to the interaction between Fe–O and OH of Dex molecules [150,151]. A band at 1071 cm^−1^ also shifted to 1026 cm^−1^ for Dex, possibly due to the occurrence of vibrational interactions between MNP and Dex molecules [150,151,152]. The Dex coating of MNPs reduced the aggregation and agglomeration of MNPs (i.e., improved dispersity), resulting in an increase in the zeta potential of nanoparticles. This was further confirmed by TEM and visually, with a greater sedimentation rate of MNPs without the Dex coating. Crystallite sizes from X-ray diffraction for MNP, MNP-Dex5% and MNP-Dex10% were determined using the Debye–Scherrer equation, assuming spherical shape (shape constant of 0.9) [153], and calculated to be 14.7 nm, 13.5 nm, and 11.5 nm, respectively. The saturation magnetization for MNP-Dex10% and MNP from the vibrating sample magnetometer (VSM) hysteresis curves was about 58 emu/g, showing the potential of NPs to be used for MRI, with similar or higher saturation magnetization when compared to other types of MNPs [154,155]. The size of the spherical DOX-loaded magnetoliposomes (DMLs) was determined to be ~100 nm (i.e., 2 times larger than plain liposomes), with a zeta potential of −3.6 mV. Compared to DOX-loaded liposomes (DLs), DMLs are more slow-releasing in terms of drug release, which could be due to the interactions between DOX and Dex-coated MNPs. Drug release studies showed that about 50% of the total DOX loaded on DMLs was released in 150 h in a sustained manner (i.e., at 37 °C) due to the stability of liposomes. The cytotoxicity in MCF-7 breast cancer cells was ~40% with treatment with DMLs and was very similar to cytotoxicity values when cells were treated with DOX only (i.e., using same concentration of 25 μM).

**Figure 1:**
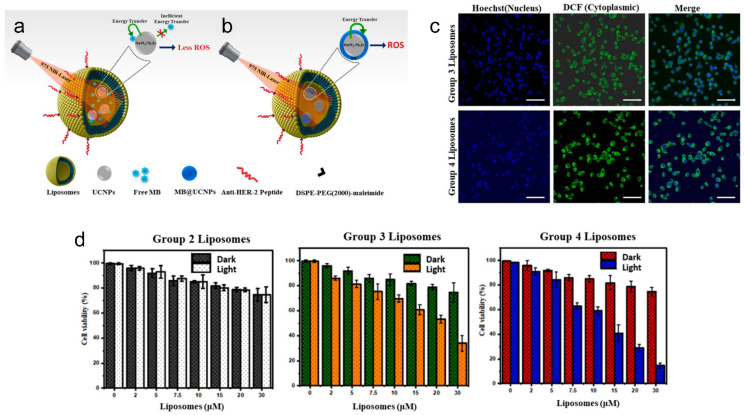
Ligand-targeted theranostic liposomes combining methylene blue attached upconversion nanoparticles for NIR-activated bioimaging and photodynamic therapy against HER-2 positive breast cancer. Illustration shows anti-HER2 peptide conjugated liposomes for selective bioimaging and PDT (i.e., using 975 nm NIR-laser) using two different combinations with LPs with free MB and UCNPs (**a**) and LPs with MB@UCNPs (**b**). Confocal images show that ROS can be generated in Group 3 LPs and Group 4 LPs using DCF-DA as a green fluorescent indicator after 5 min of laser excitation (i.e., at 975 nm) (**c**). Nuclei were counterstained with Hoechst shown in blue color. Scale represents: 50 μm. In vitro cell viability was determined using XTT assay to assess the SKBR-3 cell viability with different concentrations of LPs Groups (0–30 μM) and 975 nm NIR laser excitation for 5 min (**d**). Group 2 LPs represent LPs with UCNPs, Group 3 LPs represent LPs with free MB and UCNPs, and Group 4 LPs represent LPs with MB@UCNPs. Data are presented as mean ± SD (n = 3). Reprinted from *Journal of Luminescence* from [156], Copyright (2021), with permission from Elsevier.

Liposomes can also be constructed for active targeting, combining methylene blue (MB) attached upconversion nanoparticles (i.e., NaYF_4_:Yb, Er UCNPs) for NIR-activated bioimaging and PDT against HER-2 positive breast cancer [156]. The UCNPs can act as an upconverting energy source for the photosensitizer dye, methylene blue (Figure 1a) [157,158,159,160,161,162]. Upon NIR laser irradiation of MB@UCNPs, ROS are produced, which can be used for damaging important biological molecules (e.g., DNA, RNA, proteins) and causing cancer cell death (Figure 1b) [163]. The NPs can specifically target HER-2 positive breast cancer cells by loading anti-HER2 peptides for selective tracking and high cell penetration capabilities [164]. TEM images revealed that the encapsulation of UCNPs was within the hydrophilic core of liposomes. The size of MB@UCNPs was determined to be 15 nm using dynamic light scattering (DLS), with the size of liposomes containing MB@UCNPs being ~77 nm (i.e., LPs). Anti-HER2 peptide attachment on PEGylated LPs (i.e., LPs + DSPE-PEG(2000) maleimide + anti-HER2 peptide) further increased the size of particles to ~91 nm (i.e., with a zeta potential of −18 mV). The amount of MB released from LPs at highly acidic conditions (i.e., 3.5, 4.5) was higher compared to that at physiological pH (i.e., 7.5), showing that the LPs can function as controlled release delivery vehicles, releasing cargo upon late endosomal acidification (i.e., after endocytosis of LPs in cancer cells). The amount of fluorescence in cells from LPs with peptides and UCNPs was significantly greater than the signals from LPs with UCNPs only. To determine the type of nanoparticle that would produce the most ROS for cancer therapy, the energy transfer efficacy (η) was evaluated. The efficacy (η) was found to be 38% for LPs containing peptides, UCNPs and free MB (i.e., Group 3 LPs) and 57% for LPs containing peptides and MB@UCNPs (i.e., Group 4 LPs). This reveals that the energy transfer effectiveness for photoactivating the MB for ROS generation in Group 4 LPs containing peptides and MB@UCNPs is higher than that of Group 3 LPs containing peptides, UCNPs and free MB. This led to the ^1^O_2_ generation capability being lower in the Group 3 LPs, compared to Group 4 LPs. Fluorescence experiments also revealed that the fluorescence intensity of ROS fluorogenic markers (i.e., using 2′,7′-dichlorodihydrofluorescein diacetate, DCF-DA) in the Group 4 LPs was higher than the Group 3 LPs, which correlates to the enhanced ROS generated (Figure 1c). The cell viability in SKBR-3 breast cancer cells after treatment with 30 μM Group 4 liposomes and 975 nm NIR laser excitation (i.e., for 5 min) was ~15%, which was about 2 times lower than the cell viability after treatment with 30 μM Group 3 liposomes at the same experimental conditions (Figure 1d).

Other types of NPs that can be used for fluorescence imaging include graphene-oxide-supported liposomes (i.e., for phototriggered tissue visualization and tumor regression) [165]. The graphene oxide flake decorated liposomal (GOF-Lipo) nanohybrid can carry the anticancer drug doxorubicin hydrochloride (DOX·HCL) [166,167] and be functionalized with folic acid [166] for tumor bonding ability, through the use of the film hydration and extrusion method. DOX is used in NPs to examine the multi-stimuli (i.e., NIR light and pH)-triggered response of folic-acid-attached GOF-Lipo (GOF-Lipo-FA) under NIR light irradiation. The spherical red emissive GOF-Lipo has a size of 200 nm, with stability for at least a month and no premature drug release in physiological conditions. The elemental composition of the GOF-Lipo nanohybrid (i.e., containing P, O, C, and N elements) can be determined using scanning transmission electron microscope (STEM) elemental mapping. The temperature was found to increase from ~37 °C to ~54 °C for GOF-Lipo (i.e., at concentration of 2.5 mg/mL) after 5 min of NIR exposure (i.e., at 808 nm, 1 W). This reveals the promising photothermal response of the designed nanohybrid due to the uniform support of GOF with liposomes. Furthermore, the DOX-GOF-Lipo showed a ~3% release in hyperthermia conditions (i.e., 43 °C), with drug release more than 90% at the photothermal ablation temperature (48 °C), due to disintegration of nanohybrids. Due to the protonation of a functional group that causes the disintegration of GOF-Lipo, the designed DOX-GOF-Lipo nanohybrid showed about 100% drug release in late endosomal conditions (i.e., pH 2 and 4). The combined effect of the drug and heat generated from DOX-GOF-Lipo-FA during NIR exposure led to high MDA-MB-231 breast cancer cell death (i.e., more than 95%) and 4T1 breast cancer cell death (i.e., ~90%). Very high emissive intensity at the tumor site after 24 h post-injection of GOF-Lipo-FA was seen due to the high accumulation through specific receptor-ligand binding. The tumor volume stayed relatively constant over a period of 21 days from combined chemo-PTT treatment using the nanohybrid (i.e., three sets of treatment repeated after 2-day time intervals using 808 nm light for 5 min), with tumor weight (i.e., ~0.15 g) after treatment lower than that compared to chemotherapy treatment (i.e., ~0.2 g) and control (i.e., ~0.37 g). 

### 4.2. Cervical Cancer

**Figure 2:**
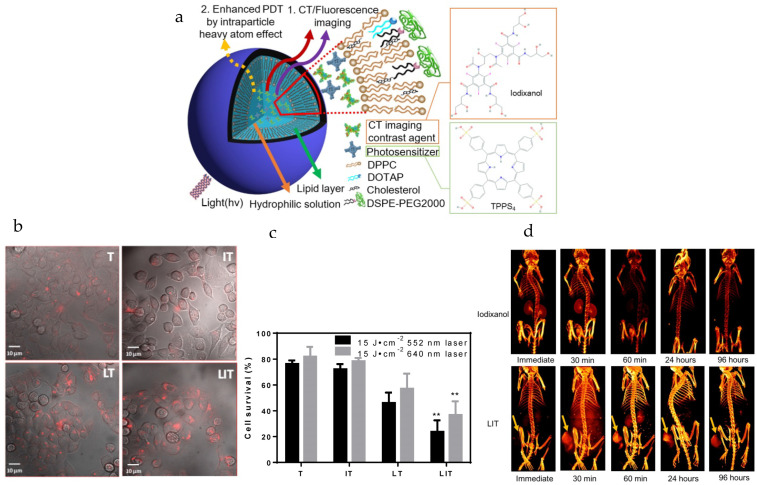
Nanoliposomes co-encapsulating CT imaging contrast agent and photosensitizer for enhanced imaging-guided photodynamic therapy of cancer. (**a**) Schematic diagram illustrates nanoliposomes co-encapsulating CT imaging contrast agent (CTIA) and photosensitizer (PS). (**b**) Optical imaging was used for visualizing cellular internalization of TPPS_4_ after 18 h incubation with free TPPS_4_ (T), free iodixanol and TPPS_4_ (IT), nanoliposomes encapsulating TPPS_4_ (LT) or nanoliposomes co-encapsulating iodixanol and TPPS_4_ (LIT). Images merging the transmission and fluorescence confocal channels are shown. Fluorescence of TPPS_4_ (red pseudocolor) was excited by a 543 nm laser. (**c**) Survival of HeLa cells was determined one day post-PDT. The dark bars indicate 552 nm laser irradiation and the light ones indicate 640 nm laser irradiation at the same irradiation dose (15 J cm^−2^). (** *p* < 0.01 compared with T, IT and LT at the same laser irradiation dose). (**d**) Three-dimensional volume-rendered images were acquired from the nude mice bearing HeLa tumor xenografts after injection of free iodixanol or NLs with co-encapsulated iodixanol and TPPS_4_ (LIT). Arrows indicate the location of the tumor. Reprinted with permission from *Theranostics* from [109]. Copyright (2019) Ivyspring International Publisher (distributed under Creative Commons Attribution (CC BY-NC) License at https://creativecommons.org/licenses/by-nc/4.0/ with no changes).

Nanoliposome (NL) co-encapsulating X-ray CT imaging contrast agents (i.e., iodixanol) and photosensitizers (i.e., meso-tetrakis(4-sulphonatophenyl)porphine, TPPS_4_) can be produced for enhanced-imaging-guided photodynamic therapy of cancer [109]. Iodinated iodixanol (Visipaque^®^) is clinically approved and used for CT imaging, while TPPS_4_ is used in concurrent CT and fluorescence (FL) imaging-guided PDT, through the use of NPs (i.e., referred to as NL co-encapsulation of iodixanol and TPPS_4_ or LIT). For enhancing singlet oxygen generation and PDT efficacy through the intraparticle heavy atom (iodine) effect on the photosensitizer (PS), the PS TPPS_4_ and iodinated iodixanol can be co-encapsulated within LIT (Figure 2a). The size of the LIT was determined to be ~117 nm, suitable for uptake by the EPR effect [168], with a zeta potential of 13.5 mV. The TPPS_4_ and iodixanol loading efficiencies in LIT were determined to be ~67% and ~64%, respectively. Compared to nanoliposomes without iodine and with only TPPS_4_ (i.e., LT), LIT formulations produced significantly higher phosphorescence signals, which corresponds to the higher amount of singlet oxygen sensitized by TPPS_4_ within LIT. This enhancement is due to increased intersystem crossing (ISC) rate in the PS molecule produced by the spin–orbit coupling enhanced by external iodine atoms (i.e., heavy atom effect) [169,170,171]. Strong fluorescence signals from HeLa cervical cancer cells were seen due to internalization of LIT (Figure 2b). The cell survival percentage of the LIT group under laser irradiation at 552 nm (i.e., using 15 J/cm^2^) and 640 nm (i.e., using 15 J/cm^2^) was measured to be ~23% and ~37%, respectively, due to the generation of ROS (Figure 2c). Fluorescence and X-ray CT signals from tumors with LIT were still strong after 96 h post-injection, with LIT mainly accumulating in the tumor (i.e., much greater signals in tumors compared to other organs). Tumor signals from LIT from CT imaging were still strong after 96 h post-injection of particles, and stronger than those from iodixanol (Figure 2d). A significant suppression of tumor growth was exhibited for the group of mice injected with LIT followed by irradiation, with the relative tumor volume being more than 6 times lower compared to other groups (i.e., LIT (no treatment), T or TPPS_4_ (irradiation), LT (irradiation)).

Multifunctional thermosensitive liposomes based on natural phase-change material can be synthesized for NIR light-triggered drug release and multimodal imaging-guided cancer combination therapy [172]. The indocyanine green (ICG)/DOX loaded and gadolinium (Gd) chelate conjugated temperature sensitive liposome nanoplatforms (ID@TSL-Gd NPs) can exhibit NIR-triggered drug release and prominent chemo-, photothermal, and photodynamic therapy properties. Due to the fluorescence, photoacoustic, photothermal and photodynamic properties of ICG [173,174,175] and magnetic properties of Gd, the NPs can be used for fluorescence/photoacoustic/magnetic resonance imaging triple-modal imaging-guided combination tumor therapy (i.e., chemotherapy, photothermotherapy, and photodynamic therapy). With the modification of folic acid (FA)-phospholipids, the accumulation of NPs in tumors can be enhanced for theranostics. In the cases of 5, 10, 20, and 40 μg/mL of ID@TSL-Gd, after 5 min of laser irradiation, the temperatures reached 45.7, 53.5, 58.5, and 61.2 °C, respectively. These temperature values are all higher than that required for irreversible apoptosis of tumor cells (i.e., 43 °C) [176]. A final DOX release of 55% could be achieved by irradiating the ID@TSL-Gd NPs in an on–off formation within 100 min (i.e., at 808 nm, 0.5 W/cm^2^), far higher than that without NIR irradiation. This stimuli response can be used to deliver greater amounts of drugs to cancer cells once the NPs have accumulated at the tumor site, while also being able to generate ROS from laser irradiation. In vitro fluorescence imaging revealed that folic acid on NPs plays a vital role in enhancing cellular uptake of imaging and therapeutic agents [176,177]. Increased cytotoxicity was seen in HeLa cervical cancer cells, induced by NIR-triggered drug release from ID@TSL-Gd (i.e., using 808 nm radiation, 0.5 W/cm^2^ for 5 min). The cell viability was as low as ~5% from treatment with the ID@TSL-Gd + laser, with a significant amount of apoptotic or damaged cells (i.e., visualized from calcein/propidium iodide staining). Furthermore, the in vivo fluorescence intensity from ID@TSL-Gd from tumors was twice the value from ICG only (i.e., after 48 h post-injection). The photoacoustic intensity and MRI signals from T_1_-weighted MR images of the tumor from the accumulation of ID@TSL-Gd peaked at 12 h post-injection. The ID@TSL-Gd + laser group showed the greatest tumor growth inhibition, compared to other groups (i.e., ICG@TSL-Gd + laser, ID@TSL-Gd, PBS + laser, PBS).

### 4.3. Brain Cancer

**Figure 3:**
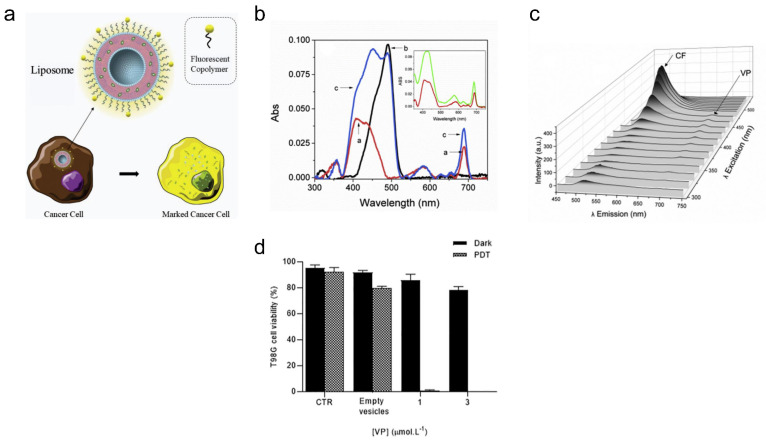
Verteporfin-loaded lipid-polymer liposomes for cancer theranostics. (**a**) Copolymer F127 modified with 5(6)-carboxyfluorescein and verteporfin can be loaded in liposomal system for cancer. (**b**) Absorption spectra of the vesicles are shown for DPPC/VP (red), F127-CF/DPPC (black) and F127-CF/DPPC/VP (blue). The insert shows the spectral overlap between the VP in methanol and in lipid-copolymer fluorescent liposomes. (**c**) The emission is shown as a function of the excitation wavelength. [DPPC] = 1.5 mmolL^−1^; [F127] = 0.015% (w/V); [F127-CF] = 0.005% (w/V); [VP] = 1.0 μmolL^−1^. (**d**) Cell viability of T98G cells was determined before and after the treatment with DPPC/F127-CF/VP. ([DPPC] = 1.5 mmolL^−1^; [F127] = 0.015% w/V; [F127-CF] = 0.005% w/V; [VP] = 1.0 μmolL^−1^). In PDT, the cell was irradiated for 20 min by a blue LED (5.52 mWcm^−2^). Incubation time = 2 h. Reprinted from *Journal of Photochemistry and Photobiology B: Biology* from [178], Copyright (2020), with permission from Elsevier.

Theranostic verteporfin-loaded lipid-polymer liposomes for photodynamic applications can be designed by a simple and fast thin-film hydration method [178]. Verteporfin (VP) is a PS that can be used for PDT and can function as a drug for the destruction of cancerous cells (i.e., with high metastatic potential) [179]. Furthermore, liposomes with the copolymer F127, modified with fluorescent probe 5(6)-carboxyfluorescein (CF), enable imaging of cancer cells (Figure 3a) [180,181]. It was found that sonication for 480 s of a solution containing 1,2-dipalmitoyl-*sn*-glycero-3-phosphatidylcholine or DPPC, F127 and F127-CF led to liposomes with the smallest size (i.e., ~88 nm) and low polydispersity index (i.e., 0.15). These liposomes were highly stable in solution (i.e., PBS) for more than a week. TEM images further confirmed the monodisperse size distribution of the liposomes, with an inner, dark and dense region representing phospholipids organized in the lipid bilayer and a lighter region representing the steric coating layer for the vesicle (i.e., from EO groups of the modified copolymer) [182]. Loading of VP in liposomes (i.e., F127-CF/DPPC/VP) increased the size from ~88 nm to ~112 nm and decreased the zeta potential from ~2.9 mV to ~1 mV, with a loading efficiency of ~93%. The absorption and emission spectra of liposomes containing F127-CF and DPPC revealed that VP is well entrapped within vesicles mostly in its monomeric state (i.e., from typical Q-band, Soret band, emission λ_MAX_ = 688 nm) (Figure 3b,c). The fluorescence quantum yield of CF in F127-CF/DPPC/VP liposomes (i.e., ϕ_F_ = 0.57) still ensures its use as a strong diagnostic tool for cancer imaging. For PDT, T98G brain cancer cells containing DPPC/F127-CF/VP liposomes were irradiated for 20 min by a blue light emitting diode (LED) at 5.52 mW/cm^2^. At a concentration of 1 and 3 μM for VP in liposomes, the T98G cancer cell viability was less than 5%, with the cell viability being greater than 75% with no light treatment (i.e., with DPPC/F127-CF/VP liposomes only) (Figure 3d). 

Transferrin(Tf)-receptor-targeted gold-based theranostic liposomes can be produced, containing both docetaxel (DCX) and glutathione-reduced gold nanoparticles (AuGSH), for brain-targeted drug delivery and imaging [183]. Transferrin is a glycoprotein used for receptor-mediated endocytosis of nanoparticles for enhancing the uptake of cancer therapy drugs such as docetaxel [184,185,186,187]. Gold nanoparticles can be used in various imaging modalities such as photoacoustic and CT imaging and due to their attractive properties, such as strong surface plasmon absorption, biosafety, stability, and ease of modification, they are ideal for both imaging and therapy applications [188,189,190]. DCX and AuGSH can be co-loaded into liposomes using a solvent injection technique and Tf post-conjugated on the surface of the liposomes using a linker (i.e., carboxylated Vit-E TPGS (TPGS-COOH)). The size, polydispersity index (PDI), zeta potential and encapsulation efficiency (i.e., of DCX) for DCX-AuGSH-TPGS-Tf (targeted) liposomes were determined to be ~270 nm, ~0.6, ~−6 mV and ~70%, respectively. A sustained mode of release was observed for about 48 h from targeted gold liposomes (DCX-AuGSH-TPGS-Tf) without any signs of burst release, which is important for reducing systemic toxicity in vivo, with surface conjugation of Tf on liposomes slowing down drug release [191]. The half maximal inhibitory concentration (IC_50_) from the treatment of C6 glioma cells with DCX-AuGSH-TPGS-Tf (targeted) liposomes was 26.82 μg/mL, much lower than IC_50_ (i.e., 42.69 μg/mL) from the treatment with DCX-AuGSH-TPGS (non-targeted) and Docel™ drug treatment alone (i.e., 79.24 μg/mL). The cell viability results that showed higher potency from the use of DCX-AuGSH-TPGS-Tf (targeted) liposomes were confirmed by fluorescence imaging, showing the greater uptake of targeted theranostic liposomes, compared to non-targeted liposomes due to receptor-mediated endocytosis (i.e., transferrin-mediated). Furthermore, the in vivo results demonstrated that targeted gold liposomes were able to deliver DCX into the brain by 3.70-, 2.74- and 4.08-folds higher than Docel™ after 30, 120 and 240 min of the treatment, respectively. Overall, the results show that the Tf-decorated AuGSH and DCX co-loaded liposomes are a promising platform for targeted nano-theranostics. 

### 4.4. Lung Cancer

**Figure 4:**
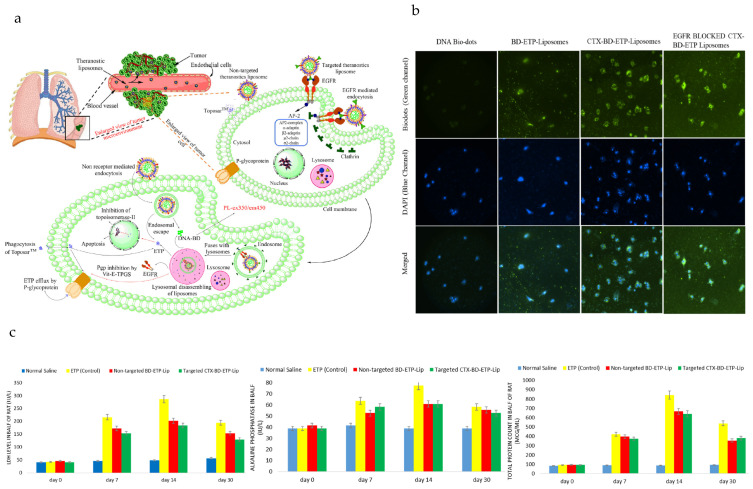
Targeted theranostic DNA-biodot-based agents for imaging and treatment of non-small cell lung cancer. (**a**) The mechanism of targeted delivery of etoposide (ETP) and DNA biodots (DNA-BD) loaded, non-targeted and targeted (cetuximab-conjugated) theranostic liposomes in lung cancer imaging and therapy is shown. (**b**) Fluorescence microscopy images were taken using A-549 adenocarcinoma cells after 48 h incubation with free DNA-BD, BD-ETP-Liposomes, CTX-BD-ETP-Liposomes and cetuximab pretreated CTX-BD-ETP-Liposomes. (**c**) Levels of alkaline phosphatase (ALP), lactate dehydrogenase (LDH) and Total Protein Count from female rats at day 0, day 7, day 14, day 30, after 7 day administration of normal saline, ETP (control), BD-ETP-Liposomes and CTX-BD-ETP-Liposomes were determined, (dose = 10 mg/kg), (n = 4). Reprinted from *International Journal of Biological Macromolecules* from [192], Copyright (2020), with permission from Elsevier.

DNA-biodot-based targeted theranostic nanoparticles can be used for the imaging and treatment of non-small cell lung cancer (NSCLC) [192]. Cetuximab (CTX) is an epidermal growth factor receptor (EGFR) inhibitor [65,193], and etoposide (ETP) is a topoisomerase-II blocker, which are loaded with DNA biodots in liposomes for targeted imaging and treatment of advanced-stage NSCLC (Figure 4a). DNA, under high pressure and temperature, condenses to form luminescent biodots (BD) [194] and can be loaded in liposomes through the solvent injection method. The particle size, PDI, zeta potential, encapsulation efficiency of ETP, encapsulation efficiency of DNA BDs and IC_50_ value (i.e., from EGFR-positive A549 lung cancer cells) of targeted CTX-BD-ETP-liposomes were determined to be ~182 nm, ~0.2, −29 mV, 70%, 42% and 0.45 μg/mL, respectively. While the particle size, PDI, zeta potential, encapsulation efficiency of ETP, and encapsulation efficiency of DNA of non-targeted particles (i.e., BD-ETP-liposomes) were very similar to those of targeted CTX-BD-ETP-liposomes, the IC_50_ value was much greater (i.e., 9.7 μg/mL) for non-targeted liposomes, due to the lack of receptor targeting towards A549 lung cancer cells. The amount and rate of ETP released from both non-targeted BD-ETP-liposomes and targeted CTX-BD-ETP-liposomes were significantly higher at acidic pH (pH 5.5) than at pH 7.4 and pH 6.4. The greater amount of ETP release at acidic pH is attributed to the increased fluidity of the bilayer membrane at lower pH due to the hexagonal phase transition of 1,2-distearoyl-*sn*-glycerol-3-phosphoethanolamine (DSPE) component in the liposomes. This can be used to improve antitumor efficacy and lower systemic toxicity as the pH at the tumor site is acidic. Images from fluorescence microscopy showed that cetuximab targeted liposomes have the highest uptake, greater than the cellular uptake of non-targeted and unencapsulated BD (Figure 4b). The cetuximab pre-treated group had less uptake than that of targeted liposomes group due to the blocking of the EGFR receptor, indicating the important role of the EGFR receptor in the cellular uptake of targeted liposomes. To determine the biocompatibility of nanoparticles, the levels of biochemical components (i.e., lactate dehydrogenase, alkaline phosphatase, total protein count) were measured (Figure 4c). The levels of all the biochemical components started to reduce with time after a period of 14 days. 

Folate-targeted paclitaxel and vinorelbine encapsulating theranostic liposomes for non-small cell lung cancer can be constructed [195]. Paclitaxel (PCX) and vinorelbine (VNB) are both chemotherapeutic agents that have been approved for the treatment of NSCLC [196,197]. Folate in nanoparticles can be used for targeting folate receptors on the membranes of cancer cells [198], while the Tc-99m radioisotope in particles can be used for SPECT/CT imaging. Tc-99m has many advantages for imaging, including its pure gamma energy and optimal half-life [199]. The size determined for liposomes with folate, PCX and VNB (Fol-Lipo/PCX/VNB) was 192 nm, with an encapsulation efficiency of 18% and 23% for PCX and VNB, respectively, and radiolabeling efficiency of 85%. Both Lipo and Fol-Lipo formulations were highly internalized by LLC1 lung cancer cells, with greater fluorescence intensity and signals from radiolabeled liposomes (targeted nanoparticles). The IC_50_ values for PCX and VNB for Fol-Lipo/PCX/VNB were determined to be 4.25 nM and 11.25 nM, respectively, and much lower (i.e., greater potency) than IC_50_ values for free PCX (i.e., 54.05 nM) and VNB (i.e., 48.91 nM). The IC_50_ values for each drug in Fol-Lipo/PCX/VNB were ~6 times lower than IC_50_ values for liposomes, with each drug only (i.e., Fol-Lipo/PCX, Fol-Lipo/VNB). The tumor uptake of ^99m^Tc-Fol-Lipo/PCX/VNB was significantly enhanced with time, and was found to be higher than the tumor uptake of ^99m^Tc-Lipo/PCX/VNB at 24 h post-administration. The Fol-Lipo/PCX/VNB formulation significantly inhibited tumor growth with a much greater weight change in tumor bearing mice, compared with the control (Fol-Lipo) and free drug combination groups (i.e., containing the same amount of PCX and VNB as in liposome formulations). These results were further confirmed by micro-CT images obtained from mice before and after the treatment, with therapeutic evaluation from measurements of tumor size. 

### 4.5. Prostate Cancer

Liposomes can be developed for paclitaxel-potentiated photodynamic theranostics, for synergistic tumor ablation and precise anticancer efficacy monitoring [200]. The dual-functional theranostic photosensitizer (PS), TPCI, possesses a very high ^1^O_2_ quantum yield of ~99% in water and can simultaneously self-report the PDT therapeutic response from the beginning of the treatment [201]. TPCI along with a first-line chemotherapy agent (i.e., paclitaxel, PTX) [202,203,204] can be co-encapsulated in liposomes for a synergistic anticancer effect against a series of tumor cell lines, including those of prostate cancer. TPCI/PTX@Lipo (i.e., liposomes with TPCI and PTX) are spherical with an average size of 100–120 nm and encapsulation efficiency of 89% for PTX and 87% for TPCI. In addition, TPCI/PTX@Lipo were stable for at least 2 weeks, in terms of size, with no obvious aggregation or precipitation. The cumulative release values of PTX from PTX@Lipo and TPCI/PTX@Lipo were 80% and 92% after 48 h, respectively, with similar drug release kinetics for PTX and TPCI from TPCI/PTX@Lipo in release media with different pH values (7.4 and 6.5). This suggests that the release of agents from TPCI/PTX@Lipo is independent of the microenvironment. Since the red fluorescence of BODIPY C11 (i.e., a detector of ROS) decreased drastically with the irradiation time (i.e., at 460 nm excitation, 4 mW/cm^2^) in the presence of TPCI@Lipo or TPCI/PTX@Lipo, the liposomes were very efficient in generating ROS. Upon irradiation at 460 nm and 1 mW/cm^2^ for 10 min, the red fluorescence from BODIPY C11 in PC3 cells pretreated with TPCI/PTX@Lipo was dramatically weakened, suggesting excellent ROS generation efficiency. The IC_50_ values of PTX in TPCI/PTX@Lipo in treating various cancer cells (i.e., at 460 nm, 1 mW/cm^2^, 10 min) were more than 9 times lower than the IC_50_ values of PTX in PTX@Lipo, and more than 4 times lower than IC_50_ values of TPCI in TPCI@Lipo, due to the effect from PDT and PTX, respectively. The combination index (CI) values of TPCI/PTX@Lipo were below 0.5 for all examined cell lines (i.e., PC3, EJ, J82, UMUC3, MCF-7), suggesting significant synergism [205,206], from the combined PDT and chemotherapy. For the PC3 prostate cancer cells treated with PTX@Lipo, the live cells were over 80% regardless of irradiation. However, after the cells were treated by TPCI/PTX@Lipo and irradiation, only 26.9% of the total cells remained alive, suggesting an extremely strong synergistic effect in killing cancer cells. After being incubated for 12 h, the fluorescence of TPCI from TPCI/PTX@Lipo in PC3 prostate cancer cells overlapped with that of Lysotracker (i.e., labelling lysosomes), suggesting effective endocytosis of the liposomes. The fluorescence of lysosomes in PC3 cells attenuated as the scanning time increased due to ROS-mediated lysosomal rupture, with the released TPCI translocated from the cytoplasm to the nucleus during the cell death process. Due to treatment with TPCI/PTX@Lipo and irradiation, the expression of the protein that pumps PTX out from the cytoplasm (i.e., P-gp) was downregulated and cleaved PARP (i.e., biomarker of cell apoptosis) [207] expression was upregulated. The PC3 cells treated with TPCI/PTX@Lipo and blue light irradiation showed the highest expression of anti-apoptotic proteins (i.e., Bcl-XL and Bcl-2) and the lowest expression of pro-apoptotic proteins (i.e., Bax and Bad), suggesting that irradiated TPCI/PTX@Lipo induces PC3 cell apoptosis through the mitochondrial pathway [208]. With the proceeding of the treatment, TPCI can be released from the liposomes and enter the nuclei during the cell death process. After binding with chromatin within the nuclei, strong fluorescence is emitted from TPCI [209,210]. The fluorescence of TPCI in PC3 cells treated with TPCI/PTX@Lipo increased 2.6-fold after irradiation, occurring concurrently with cell death (i.e., during therapy). TPCI in TPCI/PTX@Lipo can also report the cell death triggered by PTX as the TPCI fluorescence in cells pretreated with TPCI/PTX@Lipo was 2.8-fold higher than that in cells pretreated with TPCI@Lipo. Enhanced fluorescence at the injection sites of mice that received irradiation was seen, implying that tumor cells could be damaged severely, with the un-irradiated group exhibiting a negligible change in fluorescence. The results show that TPCI is effective in reporting the cell death triggered by chemotherapy, with tumor weights after treatment (i.e., from TPCI/PTX@Lipo + laser) being more than 2 times smaller, compared to other groups (i.e., saline, PTX@Lipo, TPCI/PTX@Lipo, TPCI@Lipo + laser).

**Figure 5:**
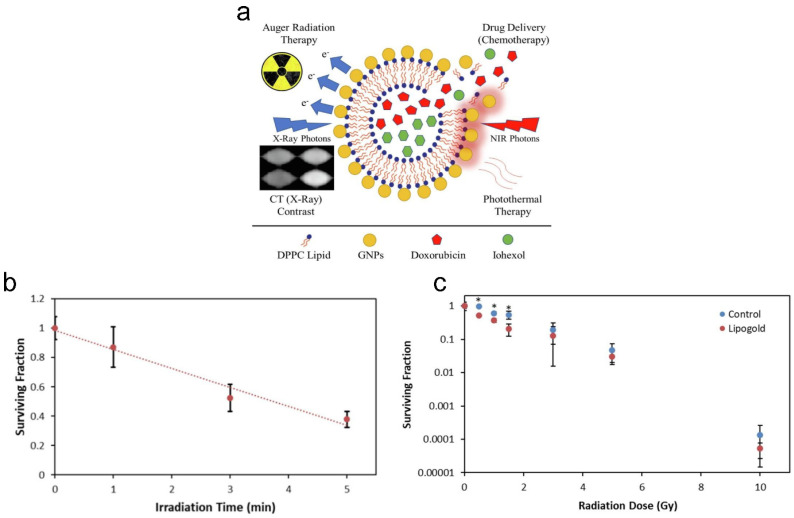
Multifunctional gold liposomes as a theranostic platform for image-guided radiotherapy. (**a**) Gold nanoparticle-coated liposomes (i.e., Lipogold) as an all-in-one platform for cancer therapies. (**b**) Clonogenic survival of PC-3 cells was determined following photothermal therapy (PTT). (**c**) Dose survival curve was determined under different doses of 6 MV X-ray irradiation. N ≥ 5 with errors bars representing the standard deviation. * *p* < 0.05. Reprinted from *International Journal of Pharmaceutics* from [211], Copyright (2022), with permission from Elsevier.

Degradable multifunctional gold liposomes (i.e., Lipogold) as an all-in-one theranostic platform for image-guided radiotherapy (IGRT) can be synthesized for prostate cancer [211]. Both doxorubicin and iohexol can be loaded in the core of liposomes, as chemotherapeutic and computed tomography (X-ray) contrast agents, respectively (Figure 5a). The ratio of gold to ascorbic acid was varied, producing a variety of blue-green-colored nanoshells. Including more gold resulted in more intense and red-shifted plasmons, which enables longer wavelengths of light to be used for penetrating the tissue deeper for theranostics. The size of gold-containing liposomes (Lipogold) was determined to be around 100 nm, with a PDI of ~0.3. Lipogold alone is largely non-toxic, although oxidative stress following uptake may occur and impact the radiation response. After 5 min of irradiation (i.e., at 808 nm, 1500 mW/cm^2^), photothermal therapy alone was sufficient to kill ~62% of PC-3 prostate cancer cells as determined via the clonogenic assay (Figure 5b). The irradiation time of 5 min was sufficient to fully release encapsulated DOX from the Lipogold due to degraded/burst nanoshells, with an increase in the temperature from ~25 °C to ~65 °C. During CT scanning, encapsulation was found to significantly improve the contrast effect of the Lipogold through the addition of both gold and iodine. A two-phase pattern was seen in the release profiles of Lipogold, with a fast release of the entrapped iohexol (i.e., ~20–30% within the first 6 h) before a slower leakage of the remaining amount. Similar release profiles have been previously seen for other liposomes with both iohexol and iodixanol [212,213,214,215]. Lipogold was found to be a highly effective radiosensitizer under low doses of radiation, with the largest decrease in survival of PC-3 prostate cancer cells (i.e., ~2.65-fold difference compared to control) observed at a 1.5 Gy radiation dose (i.e., of 6 MV X-ray irradiation), with a surviving fraction of ~0.25 (Figure 5c). 

### 4.6. Skin Cancer

Theranostic liposomes (i.e., ROS-responsive liposomes, Lipo@BODIPY11) with stimulus sensing and controlled drug release properties can be fabricated for skin cancer [216]. Upon the addition of hydrogen peroxide (i.e., reactive oxygen species, H_2_O_2_), a significant increase in FL515 (i.e., fluorescence at 515 nm) of Lipo@BODIPY11 along with a fluorescence decline at 595 nm was observed, due to the ROS-induced oxidation of the diene between the BODIPY chromophore and the phenyl group. With the increase in concentration of H_2_O_2_, the FL595 intensity increased slightly at first and then significantly dropped off for concentrations between 1 and 10 mM for H_2_O_2_. However, FL515 intensity continuously grows with H_2_O_2_ concentration. The trends seen in fluorescence intensity at low concentrations of H_2_O_2_ might possibly be due to the oxidation-induced fluorescence dequenching of C11-BODIPY (581/591) on liposome bilayers. Liposomes with a higher degree of functionalization showed a stronger tendency to undergo ROS-induced detachment of C11-BODIPY (581/591). However, over-modification (i.e., >10%) leads to the formation of undesired C11-BODIPY (581/591) aggregates, which can influence the ROS-responsive behavior of Lipo@ BODIPY11. The shifts of green FL and red FL from cytometry experiments referred to the ROS sensing and the corresponding drug release behavior (i.e., of mitoxantrone, MXT) of liposomes (i.e., Lipo@BODIPY11&MXT), respectively. The time-dependent elevation of fluorescence intensity on both channels (i.e., FL_530_ and FL_610_) was observed, due to the increased internalization level (i.e., in KB cells) or the ROS-triggered colorimetric response. Quantitative analysis revealed about a three-fold (i.e., 0.91–3.01) increase in FL_530_/FL_610_ from Lipo-FA@BODIPY11 (i.e., folate containing) after H_2_O_2_ treatment. On the other hand, the ROS scavenger, N-acetyl-L-cysteine (NAC), dramatically suppressed the H_2_O_2_-induced change in FL_530_, FL_610_ and FL_530_/FL_610_. Furthermore, there was a concentration-dependent cytotoxicity to KB cells from MXT-loaded Lipo-FA@BODIPY11, with IC_50_ lower than 10 μM MXT. The cytotoxicity of Lipo-FA@BODIPY11&MXT was enhanced upon H_2_O_2_ treatment. However, Lipo-FA@MXT without C11-BODIPY (581/591) modification displayed relatively weak cytotoxicity and showed no observable H_2_O_2_-dependent variation. Overall, the results show that the Lipo-FA@BODIPY11&MXT can be used for enhancing cytotoxicity and fluorescence imaging.

**Figure 6:**
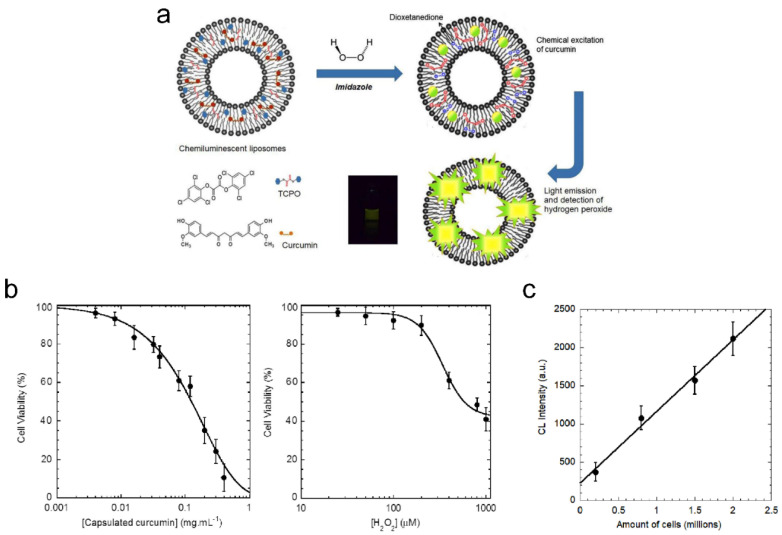
Chemiluminescent liposomes for cancer theranostics under oxidative stress. (**a**) A schematic diagram of chemiluminescent liposomes composed of peroxyoxalate and curcumin. The liposomes generate light emission instantaneously in response to hydrogen peroxide. (**b**) Relative viability values of control cells and target cells were determined after treatment with curcumin-encapsulated peroxyoxalate liposomes at different concentrations for 24 h with cytotoxicity evaluations of hydrogen peroxide on cells after 1 day. Reported values are means ± SD for three independent determinations. (**c**) The dependence of the integral light emitted during PO-CL reaction in the cells is based on their amount in the sample (y = 935.47x + 231.02, R = 0.995). Reported values are means ± SD for three independent determinations. Reprinted from *Analytica Chimica Acta* from [217], Copyright (2019), with permission from Elsevier.

Chemiluminescent (CL) liposomes as theranostic carriers can be used for the detection of tumor cells under oxidative stress [217]. The chemiluminescent liposomes are composed of peroxyoxalate (PO) [218,219] and fluorophore curcumin [220,221,222] for chemiluminescence and treatment of cancer cells, respectively (Figure 6a). The CL liposomes containing these agents can detect hydrogen peroxide using chemiluminescence intensity. Curcumin plays the dual role both as an activator in PO-CL reactions as well as an effective photosensitizer for singlet oxygen generation for PDT. The size of spherical curcumin-encapsulated peroxalate liposomes was determined to be ~170 nm, with a zeta potential of ~−9 mV. The association of curcumin with liposomes led to the peak broadening of absorption spectra of curcumin in liposomes, with a relatively small shoulder at 397 nm. The maxima shifted in fluorescence spectra from 525 nm in methanol to 515 nm after loading into liposomes, indicating that curcumin is partitioning into the vesicle. Also, the sensitized PO-CL emission was similar to the fluorescence spectrum of curcumin, under comparable experimental conditions (i.e., intensity maximum ~515 nm). These changes in the spectra confirm the singlet excited state of the fluorescent activator that formed in the CL reaction and the emitting species [218,219]. Even though experiments have revealed that the chemiluminescence intensity of free curcumin was higher (i.e., about 4-fold) than that of the encapsulated curcumin under the same experimental condition, the stability and bioavailability of lipophilic curcumin was increased by encapsulation in liposomes. The chemiluminescence intensity increased with the increasing concentration of imidazole and hydrogen peroxide, with a linear relationship between chemiluminescence intensity and concentration of H_2_O_2_ (i.e., at physiological concentrations). The viability of cells after treatment with curcumin-encapsulated peroxyoxalate liposomes at 0.4 mg/mL encapsulated curcumin was ~12%, while the cell viability was ~40% with 1 mM of H_2_O_2_ (Figure 6b). The level of ROS generation in cells with liposomes was ~28% at a concentration of 200 μM H_2_O_2_. Strong fluorescence signals were observed in melanoma cancer cells, incubated with chemiluminescent liposomes (i.e., from auto-fluorescence of curcumin). Signals from liposomes were found in the cytoplasm. Fluorescence imaging also showed that curcumin liposomes gained more efficient entry into cancer cells than free curcumin. Furthermore, the addition of chemiluminescent liposomes and imidazole to the cells under oxidative stress conditions (i.e., about 4.5 million) resulted in a detectable emission of light, while the intensity of light depended on cell density (Figure 6c).

**Table 3:** Liposomes for cancer therapy and imaging in different clinical phase trials.

Type	Therapeutic/Imaging Agent(s)	Cancer(s)	Phase(s)	Remarks	ClinicalTrials.gov Identifier	Reference(s)
liposomal doxorubicin	doxorubicin	metastatic breast cancer (MBC)	phase II	Results from clinical trials revealed liposomal doxorubicin is well tolerated and has activity similar to weekly docetaxel.	NCT00193037	[223]
pegylated liposomal doxorubicin (PLD)	bevacizumab doxorubicin	metastatic breast cancer (MBC)	phase II	Combination of bimonthly PLD and antibody bevacizumab led to modest activity for the treatment of MBC.	NCT00445406	[224]
nanoliposomal irinotecan (nal-IRI, MM-398)	irinotecan 5-fluorouracil (5-FU) leucovorin (LV) oxaliplatin	metastatic pancreatic cancer	phase I/II	The nal-IRI with oxaliplatin, 5-FU and LV (NALIRIFOX) was tolerable and generally manageable for patients with locally advanced/metastatic pancreatic ductal adenocarcinoma (mPDAC).	NCT02551991	[225,226]
liposome entrapped paclitaxel easy to use (LEP-ETU)	paclitaxel	many different advanced cancers	phase I	LEP-ETU is well tolerated and safe at doses below 225 mg/m^2^ and showed bioequivalence with paclitaxel formulated with polyethoxylated castor oil.	NCT00080418	[227,228]
DsiRNA lipid nanoparticle for MYC oncogene silencing (DCR-MYC)	double-stranded RNA	liver cancer	phase Ib/II	The lipid particles can inhibit cancer cell growth by targeting oncogene MYC.	NCT02314052	[229]
cationic liposome-DNA complexes (JVRS-100)	plasmid DNA complex	leukemia	phase I	JVRS-100 can be used for immunotherapy for eliciting cytokines important in mediating host defense against cancer.	NCT00860522	[230]
thermosensitive liposomal doxorubicin (ThermoDox^®^)	doxorubicin	liver cancer	phase III	There is a therapeutic benefit from combining thermosensitive liposomal doxorubicin with radiofrequency ablation (RFA).	NCT00617981	[231]
^99m^Tc-labeled, pegylated liposomal doxorubicin (Caelyx^®^, PLD)	technetium-99m (^99m^Tc) doxorubicin cyclophosphamide trastuzumab	many different advanced cancers	phase II	PLD and cyclophosphamide ± trastuzumab (antibody) followed by docetaxel is highly active in patients with planar gamma scintigraphy images showing accumulation of particles.	NCT01206881	[232,233]
PEGylated liposomal doxorubicin targeted against HER2 (MM-302)	copper-64 (^64^Cu) doxorubicin	advanced breast cancer	phase I	High ^64^Cu-MM-302 deposition was associated with more favorable treatment outcomes using radiotherapy and PET imaging.	NCT01304797	[234]

## 5. Future Directions

In order to achieve significant progress/advancement in the development of liposomes for cancer theranostics, some key improvements are required. The intrinsic characteristics of agents (e.g., for PTT and PDT) loaded in liposomes need to be optimized for higher efficacy, greater tissue penetration and effectiveness against tumor microenvironments. As research progresses in nanoparticle development, greater emphasis will be placed on nanoparticles that respond specifically toward the tumor environment (e.g., specific pH or proteins/enzymes expressed at high levels in tumor regions). To reduce the limitations present for imaging modalities and treatment techniques, the identification of more imaging and therapeutic agents that can be loaded in liposomes is required for combined therapy and imaging. This will enable image-guided therapy and theranostics of a wide variety of complex tumors. In addition, targeted theranostics and nanomedicine tailored according to an individual’s genetic profile will be important for clinical translation, as tumor properties governing recurrence vary based on factors such as age, race and ethnicity. 

## 6. Conclusions

Nanoparticles are promising vehicles for theranostics, having the ability to deliver both therapeutics and imaging agents in cancer cells. Encapsulation in liposomes enables greater accumulation of theranostic agents in tumors, while reducing the risk of significant damage in healthy tissues (i.e., high when using therapeutic molecules alone). Liposomes are biocompatible and biodegradable and can be modified to carry a variety of molecules for different treatments and imaging modalities (i.e., through image-guided therapy). This provides the ability of liposomes to treat different types of cancers at different levels of severity, preventing the recurrence of tumors by destroying a significant amount of cancer cells. Many of the different liposomes developed for cancer theranostics show promise, potentially leading to breakthroughs in treating more complex forms of cancer that otherwise could not be treated successfully through conventional treatments (e.g., using chemotherapy and radiotherapy).

## Figures and Tables

**Table 1:** Advantages and disadvantages of different cancer therapies.

Treatment	Advantages	Disadvantages
chemotherapy	cytostatic and cytotoxic abilities	acute side effects and high risk of recurrences
gene therapy	low risk of immunogenicity and high stability when loaded in NPs more direct inhibition of cell division of tumor cells	can cause changes to healthy genes due to off-targeting even at low doses, which can have generational effects
immunotherapy	fewer side effects because the therapy only targets the immune system immunological memory can reduce recurrence of cancer	effective for only certain cancers with high expressions of immunosuppressive proteins
photothermal therapy	strongly localized heating high tumor destruction efficiency using high temperatures within short periods of time	primarily used for superficial malignant tumors
photodynamic therapy	increased tissue penetration depth low light fluence rate increases selective apoptosis of tumor cells	tissue oxygenation is important for photodynamic effect photosensitivity reactions due to the use of PSs
magneto-thermal therapy	toxicity limited and side-effects reduced due to low doses of nanoparticles deep tissue penetration for treatment of malignant tumors	less control of local tumor temperature and greater damage to healthy tissue due to size of coils
ultrasound responsive therapy	deep tissue penetration with real-time imaging capability due to readily available ultrasound imaging systems	the different types of NPs/bubbles are limited due to the acoustic properties required
radiotherapy	lowers risk of recurrence and distant metastases due to deeper penetration of ionizing radiation	radiotoxicity due to the inclusion of RSs and ionizing radiation

**Table 2:** Overview of different imaging techniques used in cancer theranostics.

Technique(s)	Penetration Depth	Advantages	Limitations
PET/SPECT	no limit	non-invasive, high sensitivity and can carry out quantitative analysis	exposure to ionizing radiation and relatively low spatial resolution
CT	no limit	high contrast and spatial resolution	relatively high dose of ionizing radiation and exposure to ionizing radiation
MRI	no limit	non-invasive and high spatial resolution	relatively low sensitivity, high cost and slow acquisition and post processing
OI	<1 cm	non-invasive, no harmful effect by non-ionizing radiation, multicolor capability, and fast acquisition and post processing	relatively low spatial resolution
USI and PAI	millimeters to centimeters	non-invasive, real time, low cost, and no harmful effect by non-ionizing radiation	limited spatial resolution
